# Alert System to Detect Possible School-based Outbreaks of Influenza-like Illness

**DOI:** 10.3201/eid1702.100496

**Published:** 2011-02

**Authors:** Pamela Mann, Erin O’Connell, Guoyan Zhang, Anthoni Llau, Edhelene Rico, Fermin C. Leguen

**Affiliations:** Author affiliations: Florida Department of Health, Miami, Florida, USA (P. Mann);; Miami-Dade County Health Department, Miami (E. O’Connell, G. Zhang, A. Llau, E. Rico, F.C. Leguen)

**Keywords:** Viruses, respiratory infections, influenza, schools, surveillance, syndromic surveillance, outbreaks, expedited, dispatch

## Abstract

To evaluate the usefulness of school absentee data in identifying outbreaks as part of syndromic surveillance, we examined data collected from public schools in Miami-Dade County, Florida, USA. An innovative automated alert system captured information about school-specific absenteeism to detect and provide real-time notification of possible outbreaks of influenza-like illness.

Information about school absenteeism is commonly used as part of syndromic surveillance for detecting disease outbreaks in the United States. For example, health officials from the New York City Department of Health and Mental Hygiene evaluated school absentee percentage data for 2001–02 and identified moderate increases in influenza-associated absenteeism (*1*). However, absence is not always related to illness; thus, understanding why students miss school can be difficult because specific reasons are not usually recorded (*2*).

The Miami-Dade County Health Department (MDCHD) is Florida’s largest county health department and serves the Miami metropolitan area of ≈2.5 million persons. Approximately 350,000 students are enrolled in 436 schools in the Miami-Dade County Public Schools system (MDCPS), which includes public, charter, vocational, and alternative schools. Each school is required to enter students’ attendance information daily into an MDCPS database, the Automated Student Attendance Recordkeeping System. MDCHD has access to this database through a secure file transfer protocol that provides file access over a reliable data stream. Since 2007, MDCHD has automatically received these electronic raw data that contain students’ demographic and geographic information, which includes gender, race/ethnicity, age, school code, and ZIP code (*3*). After the emergence of pandemic (H1N1) 2009 virus in April 2009, MDCHD designed an automated school-based absentee surveillance system (SBASS) at the beginning of the 2009–10 school year. This system had an alert function to monitor trends in absentee activity and potentially link absenteeism with influenza outbreaks. We assessed this innovative SBASS as an adjunct to traditional disease reporting.

## The Study

We evaluated absentee data for MDCPS during September 8–October 21, 2009. Eighty-seven charter and special education schools were excluded because of consistently unstable absenteeism levels. On the basis of MDCPS’s previous year’s mean of 4.9% absenteeism, we used 8.0% as the threshold level (*4**,**5*). The mean and standard deviation were estimated in countywide and individual school levels. Alerts were automatically generated 1) for an absentee rate >8% and 2) when the percentage was at least 1.0 SD beyond the mean of the previous 30 days in countywide or an individual school, compared with their own value. At 1.0 SD, a warning was signaled, and when the standard deviations were 1.96 and 2.58 beyond the mean, yellow and red alerts were triggered, respectively. These cutoffs were set to alert at the 95th and 99th percentiles, assuming the percentage of absenteeism was normally distributed.

Only the yellow and red alerts were applied to countywide absenteeism trends by age group; however, all alerts were applied to the individual school trends. Combining absentee rates with an alert status helped exclude schools with percentage of absenteeism >8% without an alert that, on the basis of historical data, typically have high absentee rates because of low attendance. SAS version 9.13 (SAS, Cary, NC, USA), Visual Basic (Microsoft, Redmond, WA, USA), and ArcGIS 9.3 (www.esri.com) were used to design an SBASS that created 4 reports. These comprised 1) a figure with the percentage of countywide absentee trends by age group (<5, 6–11, 12–14, 15–17, and >18 years of age); 2) a table with the countywide absentee percentages by mean, ratio, standard deviation, and alert status (red alert, yellow alert, or warning); 3) a list of the alerted schools; and 4) a geographic information system map with the alerted school locations.

The applied epidemiology and research team of the MDCHD Epidemiology, Disease Control and Immunization Services (EDC-IS) performed daily school absentee surveillance. Protocol dictated contacting attendance offices when the system detected an alert. The school calendar was used to ascertain dates that would have high absenteeism because of teacher planning days, early release days, holidays, and other events. Daily school absentee reports were sent to MDCPS offices through email.

If clustering of influenza-like illness (ILI) was identified, MDCHD initiated an investigation. ILI clusters were deemed outbreaks when >2 students or staff with a clear association, such as classmates or sharing of similar activities, had symptoms of fever along with cough or sore throat within a specified period.

In our report, passive surveillance and direct reporting refer to school-initiated reporting of public health events to MDCHD. Direct reporting comprises public and private schools; the SBASS comprises public schools only.

The SBASS gave 61 red alerts, 28 yellow alerts, and 67 warnings during the study period (Table). After active investigation, 9 of 89 alerted schools were confirmed to have influenza outbreaks, and 71 persons with ILI were identified (Figure). Two of these 9 schools had simultaneously initiated reporting of outbreaks directly to MDCHD. Additionally, MDCHD received reports of suspected ILI activity from 24 public schools, none of which were confirmed outbreaks. Thus, 2 (8%) of 26 schools that directly reported to MDCHD had confirmed ILI outbreaks. Regardless of how ILI outbreaks were detected, all were investigated in accordance with EDC-IS protocol.

**Table:** Influenza-like illnesses identified through an SBASS, Miami-Dade County, Florida, USA, September 8–October 21, 2009*

Week	Dates	No. red alerts	No. yellow alerts	No. warning alerts	No. schools with outbreaks identified through SBASS	No. ILI identified through SBASS
1	Sep 8–Sep 11	3	2	17	0	0
2	Sep 14–Sep 18	8	2	16	1	27
3	Sep 21–Sep 25	9	11	10	2	17
4	Sep 28–Oct 2	9	4	7	0	0
5	Oct 5–Oct 9	16	4	11	2	7
6	Oct 12–Oct 16	16	5	6	1	20
7	Oct 19–Oct 21	0	0	0	3	0
Total†	Sep 8–Oct 21	61	28	67	9	71

*SBASS, school-based absentee surveillance system. †3 d were excluded due to school closures. October 20–21, high schools were excluded for participation in Florida’s Comprehensive Assessment Test and only elementary schools were counted.

**Figure:**
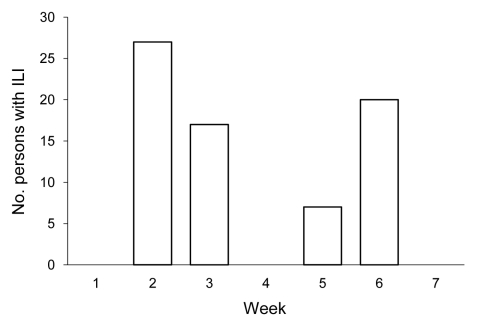
Epidemic curve of persons with influenza-like illness (ILI) identified through a school-based absentee surveillance system, Miami-Dade County Public Schools, Miami, Florida, USA, September 8–October 21, 2009.

## Conclusions

The SBASS detected all influenza-related outbreaks among public schools and proved useful in conjunction with traditional surveillance methods. Pandemic (H1N1) 2009 was a novel disease with unknown implications; therefore, implementation of an aggressive surveillance approach was needed to better characterize and understand its public health effects, particularly among school-aged children. Schools are ideal settings for detecting influenza outbreaks, and the epidemiology of influenza has shown that children play an important role in the acquisition and spread of ILI (*6*).

As of July 13, 2009, a total of 90% of positive influenza specimens in Florida tested positive for pandemic (H1N1) 2009 virus. Subsequently, the Florida Department of Health declared that identified clusters of ILI were assumed to be pandemic (H1N1) 2009 (*7*).

The inherent design of the SBASS sets it apart from other school-absentee systems, which use only percentages to determine absentee rates. Major advantages of an SBASS include an ability to identify schools with higher than normal absenteeism. The system assesses absenteeism against a historic baseline for each school. Schools with consistently high levels alone did not trigger an alert; only schools with higher than normal levels generated alerts and required follow-up. Use of an SBASS has also helped in the development of stronger partnerships between MDCHD and the school system. Frequent communication increased public health awareness and emphasized the vital role schools play in preventing and controlling disease. Additionally, the SBASS geographic information system mapping feature enabled better detection of geographic clustering when multiple schools had alerts.

Limitations of an SBASS still include an inability to capture reasons for absenteeism and its exclusion of private school attendance information. Furthermore, manual entry on the part of schools’ attendance offices may lead to a lag in data submission time, and data may contain typographical errors. Future studies should aim to extend the study period and compare influenza trends over multiple years. Research using an SBASS to detect other infectious disease outbreaks, not only in the event of a known source as was the case with pandemic (H1N1) 2009, should also be considered.
